# Evaluation of Smart City Sustainable Development Prospects Based on Fuzzy Comprehensive Evaluation Method

**DOI:** 10.1155/2022/5744415

**Published:** 2022-05-06

**Authors:** Jingyi Xu, Rui Song, Hang Zhu

**Affiliations:** ^1^School of Marxism of Tianjin University, Tianjin 300350, China; ^2^School of Artificial Intelligence, Nankai University, Tianjin 300350, China; ^3^School of Computer Science and Engineering, the University of New South Wales, Sydney, Australia

## Abstract

In this study, the method of a fuzzy comprehensive evaluation is used to analyze the research situation of smart city development, and 20 evaluation indicator systems are selected as the original indicator database; the relevant sustainable development indicator data of prefecture-level cities in the first batch of smart city pilots are collected and normalized after processing. It is used as a sample library for correlation, reliability, validity test, weight calculation, and a benchmark value for evaluation model training. By processing the original indicator database data, the membership function of the indicator is constructed, and the fuzzy set theory is used for indicators. Classify and analyze the indicators according to the model to complete the collection of the indicator database; use the neural network model to determine the weight of each indicator in the indicator system. An indicator system and evaluation model for the sustainable development of smart city construction have been constructed. To ensure the actual effectiveness of the application of the indicator system, this study has conducted in-depth research on the influencing factors and principles of the system construction, and based on this, the design of the indicator system has been carried out. At the same time, for the determination of the sustainable development weight of the sustainable development system, the analytic hierarchy process was adopted through multiple considerations, and finally, the fuzzy comprehensive evaluation model for the sustainable development of smart city construction was constructed. The model in this study is innovative in research methods and research objects. It can provide a reference value for the development of smart cities and also help the change and innovation of the existing smart city evaluation index system.

## 1. Introduction

The city is an important carrier for the development of spiritual civilization and material civilization. Since the first city was built by mankind, the city has already symbolized the survival dream of mankind. Today, no matter what kind of social system a city has, it has become a modern home with the most advanced technology and culture, representing wealth, comfort, and cutting edge. People put many good hopes in the city and continue to study various urban development models [1–3]. People are full of expectations for the appearance of the future city, and the answer to “smart city” is undoubtedly promising. The concept of a modern smart city is mainly based on two reasons. On the one hand, with the development of the time, the city begins to play a leading role in the center of the world stage [4]. With various roles scattered in all strata, this has caused rapid urban population growth, but also brought many problems and brought potential contradictions and conflicts to the society. On the other hand, the vigorous development of information technology in recent years has provided a technological foundation for smart cities [5]. If the problems in the process of urbanization have led to the demand for smart cities, and countless smart experts have proposed the concept of smart cities, then the great development of information technology has made this demand satisfied and this concept has been realized. The smart city is a brand-new urban development model, which is produced according to the development and changes of the time. Building a smart city is not only to meet the need of the development of the time but also a choice to maximize benefits [6]. First of all, the completion of a small- and medium-sized smart city can increase the dividends available for urban development by 2.5 to 3 times, which is very beneficial to the sustainable development of the city. Second, compared to traditional city management models, smart cities can greatly improve the administrative efficiency and management level of government departments. Again, the use of fully integrated information technology in smart cities can promote the coordinated and efficient operation of all aspects of city services, making our city operation safer, more efficient, greener, and more convenient, and can help solve various urban problems [7].

The concept of early smart cities is mainly related to the operational functions, management, and planning efficiency of smart technology solutions in energy, transportation, physical infrastructure, distribution and communication networks, economic development, and service delivery. Rafique S F emphasizes that smart cities use information technology to achieve the purpose of urbanization and explains the connotation of smart cities from six aspects [8]. Rodríguez F et al. defined a focused and operable smart city and believed that a smart city is the basis of human and social capital, traditional (transportation), and modern (ICT) communication through participatory governance [9]. Investment in facilities promotes sustainable economic growth and high-quality living, and the wise management of natural resources emphasizes the role of information technology. It is believed that smart cities are through the transformation of productivity to achieve urban prosperity and benefit the people [10]. Heydari A also emphasized that smart city construction promotes urban development and benefits the people [11]. Yoldaş Y, Önen A, and Muyeen S M proposed that a smart city is a complex system with a multiparty vision [12]. Gamarra C also holds similar views. Angelidou M et al. have defined the smart city in a general way [13]. The city uses information and communication technology and innovation as a means to maintain economic, social, and environmental aspects and responds to six aspects (human, economic, governance, mobility, environment, and life) and several challenges [14]. Recently, with the development of information technology, more and more foreign scholars have begun to focus on incorporating sustainable development into the concept of smart cities, emphasizing the improvement of environmental problems through technological development [15].

Anshari M et al. evaluated 70 medium-sized cities in Europe from the six aspects of the smart economy, smart population, smart governance, smart mobility, smart environment, and smart life [16]. The research results show that the top three medium smart cities are Luxembourg City, Aarhus City, and Turku City [17]. Tawalbeh L A et al. rated nine cities in the North Sea Region of Europe by setting universities, industries, governments, learning, markets, and knowledge as the six major indicators in the evaluation of smart cities [18]. After the results of systematic data comparison, any city was not possible to achieve high scores in both “university-industry-government” and “learning-market-knowledge” [19]. Dahdouh K proposed a model for measuring the performance of smart cities and conducted empirical research using Italy and Europe [20]. Zhang Q launched the ASCIMER (Assessment of Smart Cities in the Mediterranean Region) project to meet the real challenges and maintain sustainable urban development and improve the quality of life of citizens [21]. Oussous A proposed that the evaluation of cities is indispensable for the sustainable development of cities [22].

Sorting out the relevant theories such as the connotation, characteristics, and elements of smart city construction can play a supporting role in the future research work of relevant researchers, allowing researchers to more fully and comprehensively understand the basic concepts of smart cities, which is conducive to subsequent related research that went on smoothly. By summarizing the theory of smart city construction and establishing a scientific smart city construction performance evaluation index system on this basis, it can not only understand and grasp the situation of smart city construction and find deficiencies in the process of smart city construction, but also enrich the existing smart city evaluation system construction theory that is another supplement to the smart city index system. In the context of the rapid development of smart city construction, how to test the effectiveness of smart city construction, discover the deficiencies in the construction process, and further promote city construction and development are issues to be solved. The effectiveness of smart city construction requires a complete system for evaluation. In this study, through the analysis of smart city performance evaluation theory, drawing on the research and practice of the construction of relevant evaluation indicators, a scientific and objective evaluation indicator system is constructed to evaluate the sustainable development prospects of smart cities.

## 2. Research on Fuzzy Evaluation Algorithm of Smart City Development

### 2.1. Judgment Standards of Fuzzy Algorithm

Twenty smart city evaluation index systems are selected, the index system is synthesized, then the indexes are screened, reliability test is performed on each index, then the indexes are classified, and each index of each type of factor is analyzed in detail. The network model determines the index weights and finally builds a smart city evaluation index model. The scientific principle plays a fundamental role in the construction of a smart city evaluation system. In terms of this principle, it is required that the selected evaluation indicators must be scientific and reasonable, and at the same time meets the requirements of smart cities and information construction. When selecting indicators, we must focus on the scientific and theoretical analysis of indicators and select the indicators that can fully demonstrate the essence of smart cities. In addition, we must ensure that the selected indicators are objective and accurate, and can scientifically reflect. The characteristics and achievements of smart city construction are shown.

**Figure 1:**
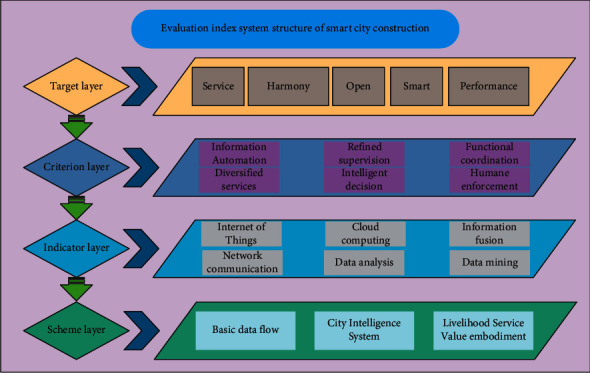
Evaluation index system structure of smart city construction.

The smart city evaluation model constructed in this study is divided into 5 dimensions and 31 indicators. Among them, there are 7 driving force indicators, 4 pressure indicators, 8 status indicators, 6 impact indicators, and 6 response indicators. The driving force index is a key indicator to promote the construction of smart cities, and the pressure index is the pressure that directly affects the construction of smart cities through the driving force. As the driving force, they are all external forces that exert on the construction process of the smart city. The difference is that the driving force exerts a hidden force, while the pressure exerts a dominant force. The state indicator refers to the state of the smart city under the pressure, mainly because the smart city meets the social needs. Impact indicators refer to the impact of smart cities on residents in turn. Response indicators refer to management measures taken against problems that arise during the construction of smart cities. The model can be divided into five dimensions: the driving force, pressure, state, impact, and response. They are an organic whole of interaction and interconnection, but the contribution of these five dimensions to smart cities is different.  Figure 1 shows the structural diagram of the evaluation index system for smart city construction.

### 2.2. Fuzzy Evaluation Algorithm

The fuzzy comprehensive evaluation method adopts the membership degree theory to transform qualitative into quantitative evaluation methods to deal with the deficiencies of unclear data and more difficult to quantify. The fuzzy comprehensive evaluation method has two types of characteristics: first, the comparison of various elements: confirm the best evaluation value, and second, according to the degree of relationship between each other, clearly define the evaluation value for various elements; second, the functional relationship: based on each the characteristics of item evaluation elements, the functional relationship between the value and the evaluation value.

#### 2.2.1. Creating Fuzzy Comprehensive Evaluation Standards

Whether the selection of evaluation criteria in fuzzy synthesis is appropriate or not will have a certain effect on whether the comprehensive assessment can be accurately and reasonably made. In the establishment of evaluation standards, the relevant documents and legal norms in the field of this evaluation standard system should be mastered from a deep perspective.

**Step 1 .**

Set up a collection of elements. The elements of the factor set are used as various elements that have a corresponding effect on the assessment object. In general, the letter U is used to indicate that the act is *U* = {*U*_1_, *U*_2_...*U*_m_}, according to *U*_*i*_ (*i* = 1, 2, ..., *m*) that represents various factors of influence. Factors in a concentrated set of factors can be unclear.

**Step 2 .**

Set up assessment sets. The various elements in the evaluation set are used as special evaluation personnel to express the conclusions of various special evaluations made by the evaluation objective research. Generally, *V* is used as the expression. In fact, *V* = {*V*_1_, *V*_2_...*V*_*n*_} and *V*_*j*_ (*j* = 1, 2, ..., *n*) can be used as a qualitative mode or a quantitative mode.

#### 2.2.2. Setting Up Weight Vector

When the fuzzy comprehensive evaluation method is adopted for evaluation, the importance of various elements is inconsistent. According to the complex weighting of elements, the purpose is to reflect the importance of various elements, and at the same time, the weight vector can be established according to the professional experience method and the AHP level analysis method. The weight of the internal elements reflects the importance of the elements. For example, the weight of the *i*-th element *U*_*i*_ is *A*_*i*_ (*i* = 1, 2, ..., *m*), so the weight set of the feature class should be *I* = (*A*_1_, *A*_2_...*A*_*m*_).

#### 2.2.3. Establishing Evaluation Matrix

Assume that the nth next *U*_*n*_ in the first category is evaluated, so the membership of the nth element in the alternative set is *r*_*nt*_ (*n* = 1, 2, ..., p; *t* = 1, 2, ..., *R*). The single element evaluation matrix of the first-level fuzzy comprehensive evaluation is as follows:

(1) $$\begin{array}{c} T_{i}=\left[\begin{array}{cccc} t_{i11} & t_{i12} & \ldots & t_{i1n}\\ t_{i21} & t_{i22} & \ldots & t_{i2n}\\ \ldots & \ldots & \ldots & \ldots \\ t_{im1} & t_{im2} & \ldots & t_{imn} \end{array}\right]. \end{array}$$

Therefore, the fuzzy comprehensive evaluation set of the factor *i* is as follows:

(2) $$\begin{array}{c} A_{i}=M_{i}T_{i}=\left(a_{i1},a_{i2},\ldots ma_{1n}\right)\left[\begin{array}{cccc} t_{i11} & t_{i12} & \ldots & t_{i1n}\\ t_{i21} & t_{i22} & \ldots & t_{i2n}\\ \ldots & \ldots & \ldots & \ldots \\ t_{im1} & t_{im2} & \ldots & t_{imn} \end{array}\right]. \end{array}$$

The correlation between the first-level fuzzy comprehensive evaluation and the second-level fuzzy comprehensive evaluation is as follows: the former is used as the single element evaluation in the latter evaluation process, and the latter is used as the single element evaluation matrix for evaluation, which is used as the former evaluation matrix:

(3) $$\begin{array}{c} T=\left[\begin{array}{c} B_{1}\\ B_{2}\\ \ldots \\ B_{m} \end{array}\right]=\left[\begin{array}{c} M_{1}B_{1}\\ M_{2}B_{2}\\ \ldots \\ M_{m}B_{m} \end{array}\right]. \end{array}$$

The relevant description of the second-level fuzzy comprehensive evaluation set is as follows:

(4) $$\begin{array}{c} M=I\cdot T=I\cdot \left[\begin{array}{c} M_{1}B_{1}\\ M_{2}B_{2}\\ \ldots \\ M_{m}B_{m} \end{array}\right]=\left(m_{1},m_{2},\ldots ,m_{p}\right). \end{array}$$

Based on the fifth equivalence of the evaluation conclusion, the evaluation conclusion matrix P can be quantified according to the graded scoring method to obtain the final calculated value. If *F* = (*f*_1_, *f*_2_...*f*_*m*_) is taken as a set of scores, as a column vector, *f*_*j*_ represents the score of the graded evaluation conclusion, and if the percentage score is adopted, the following formula is obtained. Since there is a relationship between the evaluation results of each unit in the system, the importance of each evaluation result is unbalanced, and the contribution to the safety evaluation conclusion varies, so it is best to sort out, classify, and classify the evaluation results before writing the evaluation conclusion. Results are listed in order of severity and frequency of occurrence.

(5) $$\begin{array}{c} F_{j}=100\frac{m+1-j}{m}. \end{array}$$

After obtaining the evaluation standard *Bk*, a percentage system is chosen to use to make a quantitative solution to the evaluation conclusion set *D*. It can be recorded as low risk (10, 30); low risk (30, 50); medium risk (50, 70); high risk (70, 80); and high risk (80, 100). Second, the score vector *C*^*T*^ = (*C*_1_, *C*_2_, ...*C*_*n*_) about the evaluation conclusion can be obtained, so the final value can be calculated.

(6) $$\begin{array}{c} D=\frac{1}{\sum_{i=1}^{m}B_{i}}{\text{BC}}^{T}. \end{array}$$

Because scores of various evaluation conclusions are used as an interval, the three items of the evaluation conclusion score vector combined by the upper, middle, and lower limits of each element interval are usually measured by categories and then selected.

(7) $$\begin{array}{c} W_{j}=-k\sum_{i=1}^{m}p_{ij}\ln\;p_{ij},\;k=1/\ln m,\\ W_{j}=-k\sum_{i=1}^{m}p_{ij}\ln\;p_{ij},\;k=1/\ln m,\\ U_{k}=\left\{\begin{array}{cc} \frac{w_{k}-1}{w_{k}}\text{,} & w_{k}-1\geq w_{k}\\ 1, & w_{k}-1\leq w_{k} \end{array}\right.,\\ H_{m}={\left(1+\sum_{k=2}^{m}\prod_{i=k}^{m}w_{i}\right)}^{-1}. \end{array}$$

### 2.3. Experimental Sample Data and Indicators

Through statistics of the subjective sentences in the large sample, the methods of manually extracting high-frequency words and by software are used to classify and analyze similar words, and Table 1 is obtained. Through the qualitative classification and quantitative analysis of the subjective question results in the questionnaire, the functional requirements are mainly convenient, efficient, and low cost, the word frequency is 119 times, 104 times, and 80 times, and the public is worried about enjoying efficient and convenient services. At the same time, personal information will be leaked and the security cannot be guaranteed. The high-frequency word analysis can categorize the public's expectations for smart cities into the following categories: pragmatic and rich in content (64 times), which is manifested in public services, social management, and ecological liveability services; in terms of aspects, high efficiency (104 times), convenience (119 times), and low cost (80 times) are required.

Overall, services that are close to people's livelihood in terms of content needs are the focus of public attention. In terms of service function characteristics, high efficiency, convenience, low cost, and safety have become the focus of public attention. In addition, some members of the public hope that government agencies will expand publicity so that the public can better understand and use the effects of smart cities. This open question also exactly corresponds to the content reflected in the survey of the needs of different services of smart cities. The above analysis provides an empirical basis for the establishment of the smart city indicator evaluation system in the following chapters.

Starting from the characteristics of the public's demand for smart city construction content and demand functions, the infrastructure, public services, social management, ecological liveability, and industrial system in the effectiveness index are considered as the first-level indicators of the smart city construction-level preliminary evaluation index system The middle level is to decompose the high-level indicators, and the lowest level is to expand the middle-level indicators. The preliminary index system consists of 5 first-level indicators, 15 second-level indicators, and 40 third-level indicators. The initial indicator system is shown in Table 2. Evaluation of sustainability from a social perspective is the commonality of the above five indicators, which are based on individual welfare. Their progressiveness lies in taking the ecological dimension into account, while their one-sidedness lies in the analysis of sustainability only from the point of view of human beings themselves. On the premise of economic stability, human health, and environmental protection, the quality of human life is improved; the use of nonrenewable resources should be optimized; the use of renewable resources should be sustainable; the impact of human activities on the environment, human health, and ecology should be maintained as much as possible.

The main method of implementing urban planning and the means of coordinating the organization and implementation of urban construction are comprehensive urban development. Urban planning is the unified planning and deployment of urban economic, social, technological, and cultural development plans and strategies under certain conditions. The comprehensive development of the city will be carried out by the deployment of the unified plan, and the construction of other projects such as industry, transportation, housing, science, education, culture, health, commercial services, and municipal engineering in the development area will be arranged in an overall manner to ensure that the past and the future will be comprehensively and reasonably connected.

**Figure 2:**
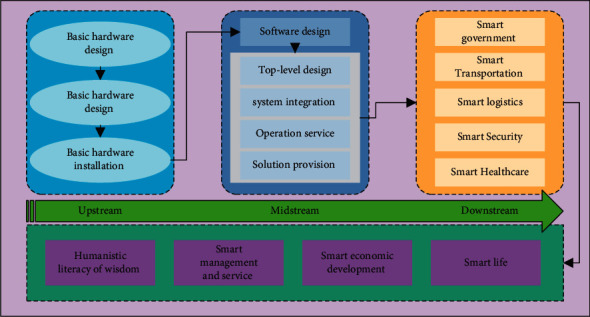
Smart city construction and operation effects.

Capability indicators refer to the basic ability evaluation indicators for the construction and operation of smart cities, that is, the basic ability evaluation indicators for cities to use various resources to build and operate smart cities. Emerging technologies such as big data, cloud computing, and spatial geographic information provide strong technical support for the construction of smart cities. Capability indicators mainly include the level of open sharing and development of information resources, network security assurance capabilities, technological innovation capabilities, and improved mechanisms. The effective index refers to the evaluation index of the construction and operational effect of the smart city and is the evaluation of the effect of the smart application. Effectiveness indicators mainly evaluate a series of qualitative or quantitative indicators such as the convenience, efficiency, and liveability of life that smart city construction brings to people's lives from the perspective of public perception. The construction and operational effect of the smart city are shown in Figure 2. The theory of comprehensive urban development and sustainable development is introduced into the new city planning; an evaluation index system is established for the sustainable development planning of the new city including five dimensions of ecology, society, economy, culture, and policy; and an evaluation method is proposed based on multifactor comprehensive evaluation method and fuzzy mathematics. The evaluation method of sustainable development planning of new cities provides a scientific, reasonable, and easy-to-operate evaluation system for the sustainable evaluation of new city planning. The evaluation indicators include five dimensions. It is worth noting that none of these indicators can be directly quantified and more focused on qualitative analysis. The evaluation objects are all over the world. Looking at the previous evaluation results, we will find that the smart community awards have covered most countries in the world. A horizontal comparison of the construction status of smart cities will show whether the construction of cities is good or bad. The degree of intelligence in the originally developed urban areas will be higher.

## 3. Results Analysis

### 3.1. Index Weight Determination Analyses

**Figure 3:**
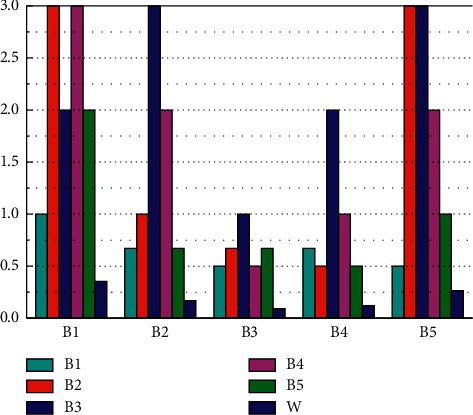
Evaluation results.

This study determines the weight of the smart city construction performance evaluation index system according to the specific steps of the analytic hierarchy process. First, the questionnaire is designed. The weight of the indicator system in this study is based on the fuzzy comprehensive evaluation method. Therefore, the expert questionnaire is designed to obtain the required data. Next, questionnaires are issued and expert questionnaires are issued to experts in five smart city-related professional fields. Then, the questionnaires of the experts are collected and distributed, the questionnaire information of the five experts is sorted out, and finally, a judgment matrix is formed and a consistency test is conducted. According to the principle of maximum membership, the performance level of smart city construction is generally “good.” From the perspective of membership, the “very bad” membership for smart city construction is 0.0112, the “bad” membership is 0.0207, the “general” membership is 0.2845, the “good” membership is 0.2714, and the “excellent” membership degree is 0.2142. The evaluation results obtained by fuzzy comprehensive evaluation analysis are shown in Figure 3.

**Figure 4:**
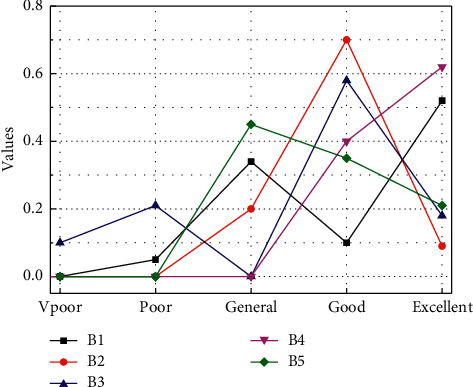
Criteria-level performance evaluation results.

From the performance evaluation results of the five main dimensions that affect the comprehensive evaluation results, the evaluation performance of the public service B1 dimension of smart city construction is “general,” the industrial modernization level B2 dimension performance is “good,” and the environmental liability level B3 performance of the dimension is “good,” the performance of the economic development level B4 dimension is “excellent,” and the performance of the urban infrastructure B5 dimension is “excellent.” It can be seen that the construction of smart cities has achieved certain results. Through the construction of information infrastructure, the city's industrial structure has been upgraded to promote the development of the city's economy, which has achieved good performance evaluation results from the level of environmental liability. The performance results show that the concept of green sustainable development has not been forgotten while developing the smart city economy. While seeing the achievements of smart city construction, it is more important to see the deficiencies in the construction process. For example, the result of the public service-level performance evaluation is “general,” which is a relatively poor aspect of the five dimensions. This may be caused by insufficient attention to social service management in the process of building a smart city. When analyzing the results of performance evaluation, it should be clear that not only the overall evaluation results should be seen, but also the specific performance evaluation should be related to the overall performance of the smart city by analyzing the evaluation results of each layer of the index system. As shown in Figure 4, it is the result of performance evaluation at the criterion level.

### 3.2. Validity Analyses

**Figure 5:**
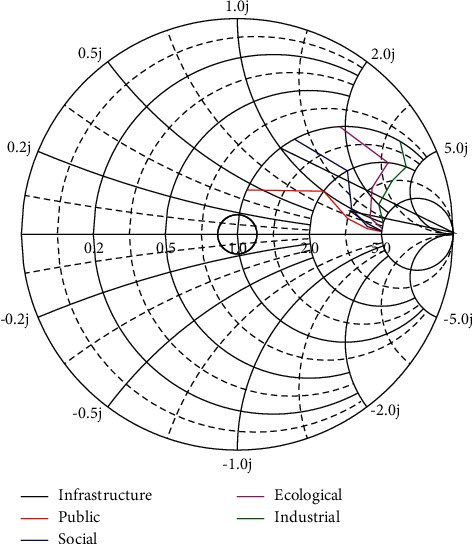
Scoring matrix.

**Figure 6:**
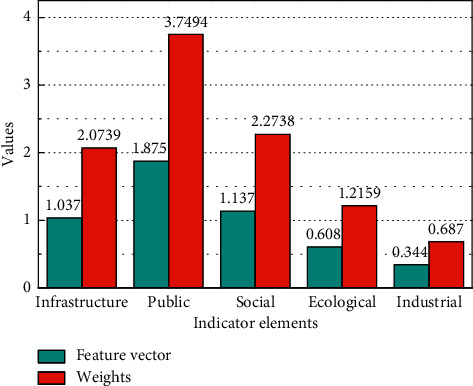
Weighted score.

Four experts in the field are invited to score the importance of the indicator elements. The weight of the four experts is 30%, 30%, 20%, and 20%, respectively. The score of the four experts is multiplied by their weight and then accumulated. The importance of indicators is scored as shown in Figure 5 with an expert scoring the first-level indicators. The weighted score is shown in Figure 6. Under open conditions, the influence of the internal factors of the system deviating from the steady state may be particularly obvious due to the interaction of factors between the internal and external environments of the system, resulting in a gradual increase in the correlation within the system, which in turn makes the system self-organizing, and evolve from low level to high level and from disorder to order in level and state, respectively. The purpose of a system means that in an organizational system that interacts with the environment, to a certain extent, it is not or less affected by empirical conditions and exhibits a certain characteristic tending to a certain predetermined state.

**Figure 7:**
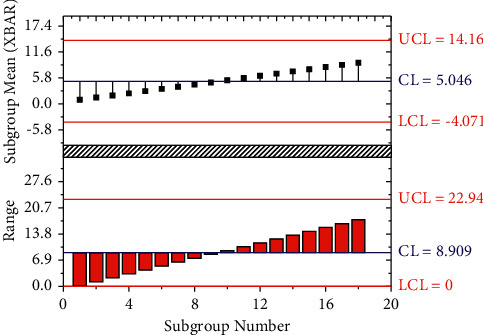
Comprehensive score.

According to the data normalization method, the original data of these 18 city-related indicators are normalized to eliminate the dimensional difference between the indicators. The normalized data are introduced into the neural network model constructed above. The input layer of the model is the 31 indicators after the previous screening. The output layer contains an indicator, that is, the comprehensive index. This item is the target output value and is set to a null value that the hidden layer contains 14 hidden nodes. The comprehensive score of the smart city development level of these 18 cities in 2019 is calculated, and the score of various indicators of 18 cities is obtained through the calculation as shown in Figure 7.

**Figure 8:**
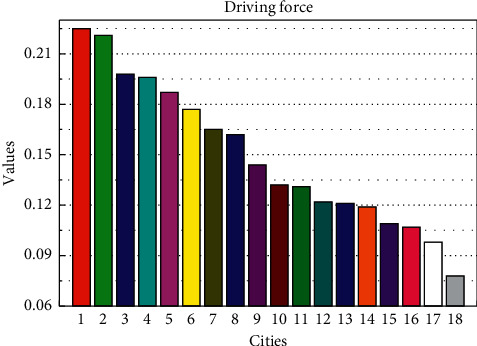
Driving force index distribution.

It can be seen from Figure 8 that it is in the top three in the driving force indicator dimension. Since the internet penetration rate occupies the main position in this indicator dimension, it can be concluded that this is inseparable from the development of a new generation of information technology without perfect infrastructure. Ranked fourth, as early as 2019, the government began planning smart city construction. After more than eight years of hard work, the overall development level is relatively balanced. In addition, as the only e-commerce capital in China, its smart city driving index is also being at the forefront and being at the bottom shows that the driving force of this smart city is insufficient. For this reason, the primary task of smart city construction is to improve infrastructure construction. In the construction of smart cities, it may not be difficult to build a logical framework. For example, from the perspective of the logical structure of smart cities, it includes smart government, smart people's livelihood, smart economy, smart infrastructure support, and smart spatial layout. From the perspective of infrastructure, no matter what kind of city you build a smart city, you cannot do without the city's data center, application platform, exchange platform, and database, relying on the internet, big data, cloud computing, mobile internet, internet of things, and other modern information technologies support.

### 3.3. Sustainability Analyses

**Figure 9:**
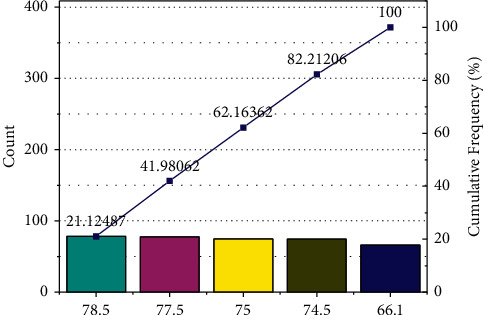
Cluster histogram of smart city construction maturity score.

The overall level of smart city construction is at a mature stage, but the scores of various subindicators are still slightly different. The score of the five implementation levels for the construction of smart cities is shown in Figure 9.

The overall level of smart city construction is relatively high, and it is in the upgrading stage, and the maturity levels of the five aspects involved in the construction level are consistent. However, for the in-depth analysis of various indicators, it is not difficult to see that the construction at each level is still steadily developing, but there are still deficiencies.

It can be seen from the above data that the infrastructure development score is 74.629, which is in the upgrading stage, including information infrastructure at 50.924 points and public infrastructure at 86.464 points. The low score of information infrastructure is mainly due to the low satisfaction of Wi-Fi coverage in public places, so government departments should strengthen the construction of information infrastructure in public places such as train stations and bus stations. Although the score of the construction of smart light poles in public infrastructure is relatively high, as far as the construction of smart light poles nationwide has been completed, 700 have been completed, which is in a leading position compared with other cities. However, according to the existing data, the coverage rate is still too low. Because retrofitting existing light poles with smart light poles will involve the reconfiguration of underground network cables and power distribution facilities, the cost of renovation is higher compared to new construction. This is also the reason that most cities do not follow in the development zones or new industrial parks, reasons for mass popularization. Therefore, government departments should overcome the difficulties and increase financial investment to improve the construction of infrastructure. The public service score is 78.040, which is in the improvement stage, including an education demand score of 82.546, medical treatment score of 81.035, transportation demand score of 71.589, and social security and employment demand score of 86.685. Among them, the traffic demand score is low, and the remaining three want to score more balanced. The traffic demand score is mainly affected by the accuracy of real-time traffic information. From the above data, it can be seen that the municipal government has invested more efforts to promote the construction of public services so that the development of all aspects is more balanced. However, according to the previous statistics, the public is more concerned about the demand for transportation at the level of public services. The proportion of transportation is relatively large, but the public's satisfaction with transportation is not high, resulting in the lowest score of public services in the five dimensions. From the above analysis, it can be seen that the current satisfaction of people with traffic problems is not high, and government departments should focus on the public's demand for fast and efficient transportation when providing public services.

**Figure 10:**
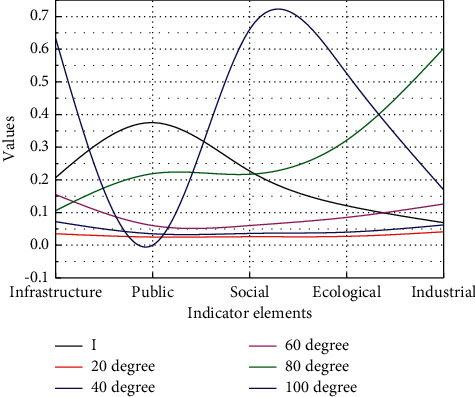
Statistics of equipment used by students to access courses.

After finding the membership of the underlying index, the information infrastructure in the secondary index is used as an example to calculate its membership. The index includes the fiber to the home rate and the public area Wi-Fi coverage satisfaction, as shown in Figure 10.

The construction content is effectively managed, and public perception is good. Among them, the infrastructure score was 74.629, the public service score was 78.040, the social management score was 78.972, the ecological livability score was 75.579, and the industrial system score was 65.938.

The main objectives of the “new smart city” mainly include the following aspects: serving the people, improving the efficiency of urban governance, promoting the opening and sharing of data, and ensuring scientific and reasonable economic development. The use of system planning, information-led, and reform and innovation measures can promote the effective combination of information technology and urban modernization so as to ensure the coordinated development of the country and the city. Influencing factors such as per capita GDP, urbanization rate, employees in scientific research, and technical services have a significant impact on the sustainable development of smart cities. The difference in per capita GDP is reduced by 0.853 units for each unit of reduction in the difference in the sustainable development of smart cities; the difference in the sustainable development of smart cities is reduced by 0.292 units for each reduction in the urbanization rate difference by one unit. Every time the difference in employees is reduced by one unit, the difference in the sustainable development of smart cities is reduced by 0.017 units. The influencing factors also have an impact on various factors of the sustainable development of smart cities. Differences in scientific research and technical practitioners have a significant impact on the differences in smart investment and construction factors for the sustainable development of smart cities. The difference in the sustainable development wisdom guarantee factor has a significant impact; the urbanization rate difference has a significant impact on the difference of the wisdom basic factor of the sustainable development of the smart city.

## 4. Conclusions

This study comes to the development status of smart cities and finds that the overall development of smart cities is booming, and various regions are also actively exploring development strategies. The original intention of the smart city is to enable every applied city to embark on a healthy and perfect sustainable road. In response to the problems arising from the sustainable development of smart cities, it should be addressed in terms of economic growth, people's problems and environmental science, urban infrastructure, and ecological environment. Targeted improvements should be awaited from different angles. It was used as a sample library and evaluation for correlation, reliability, validity test, and weight calculation, the benchmark value of model training. Then, the index data of the original index database was processed, the membership function of the index was constructed, the fuzzy set theory was used to screen the index of the original index database, and the index was classified and analyzed according to the DPSIR model. The simplification of the index database was completed, and a simplified index system was generated; the BP neural network model was used to determine the weight of each index. Through the above steps, a set of three-level, five-dimensional, and 31 three-level indicators of the smart city evaluation system model was finally constructed. Finally, this study selected 18 sample city-related data, evaluated and analyzed the construction of smart cities, verified the scientific and operability of the evaluation system, and also found problems in the construction of smart cities. Corresponding improvement strategies can better promote the healthy and sustainable development of smart cities.

## Figures and Tables

**Table 1:** Initial analysis of keyword frequency of public expectation for smart city construction.

Serial number	Keywords	Word frequency	Serial number	Keywords	Word frequency
01	Convenient	119	11	Easy employment	17
02	Efficient	104	12	Online education	55
03	Low cost	80	13	Personal information security	48
04	Information security	68	14	Expand publicity	30
05	Rich content	64	15	Network coverage	20
06	Public transit	50	16	Public experience	28
07	Government affairs	55	17	Diversified service	30
08	See a doctor	98	18	Social security service	20

**Table 2:** Preliminary evaluation index system.

First-level indicators	Secondary indicators	Third-level indicators
Infrastructure	Information infrastructure Public infrastructure Educational needs Medical needs	Fixed broadband home coverage
		Fiber to the home rate
		Satisfaction of Wi-Fi service in public places
	Traffic demand Social security and employment information service	Popularization rate of intelligent display screens at bus stops
		Smart light pole construction level
		Intersection TV monitoring points (a)
Public service	Government management Secondary indicators	Convenience and richness of obtaining educational resources online
		The rationality of the price of online platform learning resources
	Information infrastructure Public infrastructure	Satisfaction of online appointment and electronic medical record popularization
		Self-service network platform to pay medical expense satisfaction
Infrastructure	Information infrastructure Public infrastructure	The abundance of free learning resources
		One-stop satisfaction
	Educational needs	Satisfaction with the urban citizen hotline
		Satisfaction of diversified service channels

## Data Availability

The dataset can be accessed upon request.
